# Supplementary material for: Likelihood-based estimation of substructure content from single-wavelength anomalous diffraction (SAD) intensity data
Source: Acta Crystallogr D Struct Biol. 2021 Jun 18;77(Pt 7):880–93. doi: 10.1107/S2059798321004538 (PMC8251343; doi:10.1107/S2059798321004538)
Supplement: Supplementary file 1 [file d-77-00880-sup1.pdf]

STRUCTURAL BIOLOGY

Volume 77 (2021)

Supporting information for article:

Likelihood-based estimation of substructure content from single-wavelength anomalous diffraction (SAD) intensity data

Kaushik S. Hatti, Airlie J. McCoy and Randy J. Read

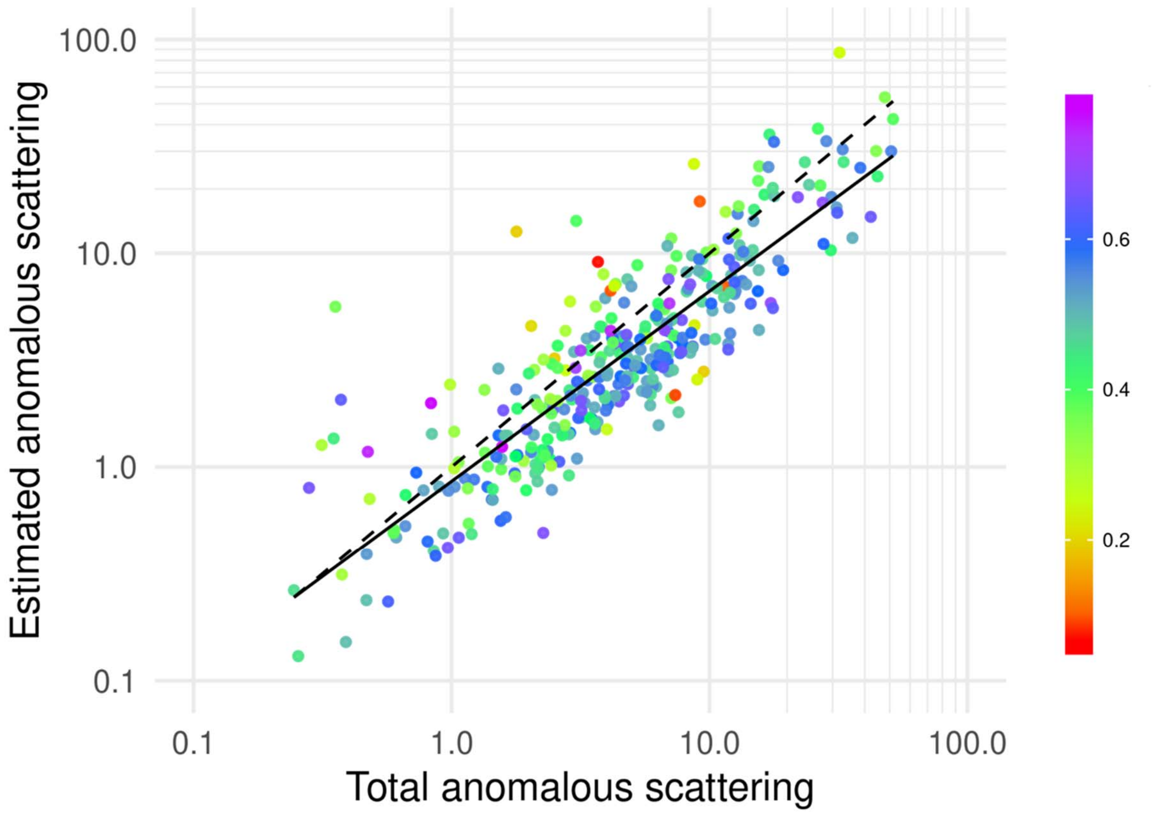

Figure S1 Estimation of equivalent fully-occupied number of primary anomalous scatterers, for data deposited as either intensities or amplitudes. The horizontal axis is the total anomalous scattering power of the gold-standard substructure (f”-weighted sum of squared occupancies of refined sites) and the vertical axis is the estimated anomalous scattering power. The dashed black line represents a perfect prediction while the solid black line shows the least-squares linear fit of the estimates. Each data point is coloured by the map correlation coefficient as shown in the legend. Both axes are plotted on a $\log_{10}$ scale.

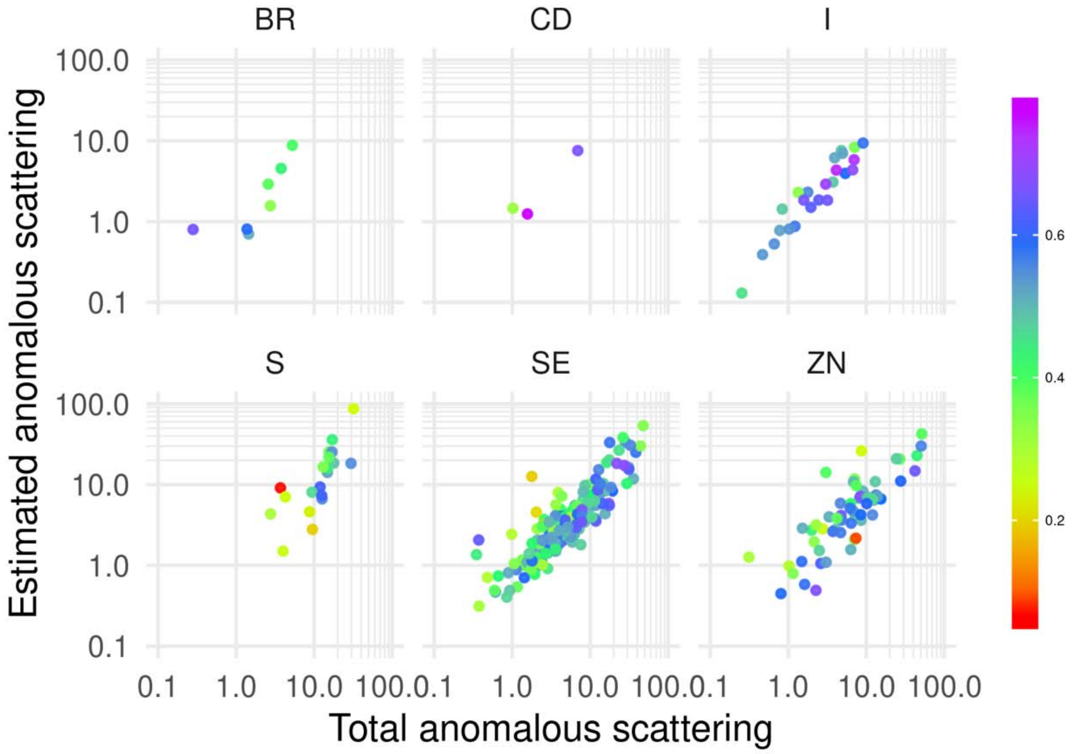

Figure S2 Estimation of equivalent fully-occupied number of anomalous scatterers for different element types. The horizontal axis is the total anomalous scattering (f''-weighted sum of squared occupancies of refined sites) and the vertical axis is the estimated anomalous scattering power. Each data point is coloured by the map correlation coefficient as shown in the legend. Both axes are plotted on a $\log_{10}$ scale. Only data for elements with at least 3 cases and over 75% contribution to the total anomalous scattering power are illustrated in this figure.
